# Persistent Organic Pollutants in Whale Shark (*Rhincodon typus*) Skin Biopsies from Bahía de Los Ángeles, Mexico

**DOI:** 10.1007/s00128-023-03841-2

**Published:** 2023-12-23

**Authors:** Stephanie Itzel Villagómez-Vélez, Elsa Noreña-Barroso, Felipe Galván-Magaña, Rogelio González-Armas, Gabriela Rodríguez-Fuentes, Ana Judith Marmolejo-Rodríguez

**Affiliations:** 1 Centro Interdisciplinario de Ciencias Marinas, Instituto Politécnico Nacional, Av. Instituto Politécnico Nacional S/N, 23096 La Paz, Baja California Sur México; 2 Unidad de Química en Sisal, Facultad de Química, Universidad Nacional Autónoma de México, Puerto de Abrigo S/N, 97356 Sisal, Yucatán México; 3 Laboratorio Nacional de Resiliencia Costera (LANRESC), Puerto de Abrigo S/N, 97356 Sisal, Yucatán México

**Keywords:** Gulf of California, Hydrocarbons, Pesticides, Skin biopsies, Whale shark

## Abstract

The whale shark (*Rhincodon typus*) is a filter-feeding organism that can be considered a sentinel species, and Bahía de los Ángeles (BLA) in the Gulf of California is an important sighting site for these elasmobranchs. This filter-feeding organism can be considered a pollutant sampler from the marine environment. Persistent organic pollutants are toxic compounds with high mobility and environmental persistence, bioaccumulation and trophic transfer. Among these are polycyclic aromatic hydrocarbons (PAHs) and organochlorine pesticides (OCPs). The present work aimed to determine concentrations of PAHs and OCPs in whale shark skin biopsies, collected in 2021 at BLA. Mean detected levels of PAHs and OCPs were 279.4 ng/g dw (dry weight) and 1478.1 ng/g dw, respectively. Analysis of similarities between the ordered sizes (4.2–7.6 m) and the concentrations of PAHs and OCPs indicated no significant differences. Individual PAHs detected indicate pyrogenic and petrogenic sources; the presence of pesticides at levels higher than those of hydrocarbons may be related to agricultural activity in the areas surrounding the Baja California peninsula. This study is the first report of PAH levels in *R. typus* for the Gulf of California and Mexico.

Persistent organic pollutants (POPs) are ubiquitous compounds in the marine environment that are resistant to degradation and potentially toxic to aquatic organisms. They are lipophilic compounds that can bioaccumulate and biomagnify throughout the food web. POPs can travel great distances from the original source by wind, water, and even migratory species (long-range transport) (EPA 2002; Chen 2022). POPs include organochlorine pesticides (OCPs), which are used to control pests and vector-borne diseases such as malaria (EPA 2002; Sparling 2016); as well as polycyclic aromatic hydrocarbons (PAHs), which are released into the environment by natural processes such as forest fires and volcanic eruptions, but also by human activities like the incomplete combustion of fossil fuels, oil spills, vehicle gas emissions, among others (Boehm 1964; Sparling 2016; Lawal 2017).

Filter-feeding elasmobranchs are recognized as long-range sentinel indicator species of the ecosystem, providing an estimate of pollutant levels in marine environments (Gerhardt 2002; Alves et al. 2022; Boldrocchi et al. 2022, 2023). The whale shark (*Rhincodon typus*) is considered the largest fish in the world and it is listed as endangered on the Red List of the International Union for Conservation of Nature (IUCN; Pierce and Norman 2016). The whale shark is a highly migratory organism. It is considered a possible sampler of the marine environment, acting as a bioindicator of pollutants at a regional level (Fossi et al. 2017; Boldrocchi et al. 2020; Boldrochhi et al. 2022). There is a growing interest in monitoring the presence of contaminants in this species, both for its potential as a bioindicator and for its conservation. Therefore, this study aimed to analyze the presence of PAHs and OCPs in whale shark skin biopsies in the sighting area of Bahía de Los Ángeles (BLA), in the Gulf of California, Mexico; which is influenced by tourist, fishing, agricultural and fuel transportation activities. A mean travel rate of up to 23.6 km/day has been reported in whale sharks tagged in BLA (Eckert and Stewart 2001), but *R. typus* juveniles can remain in BLA from several days to a month or more (Ramírez-Macías et al. 2012); therefore, subacute exposure to these contaminants could occur in the study area (ATSDR 2015).

## Materials and Methods

**Fig. 1:**
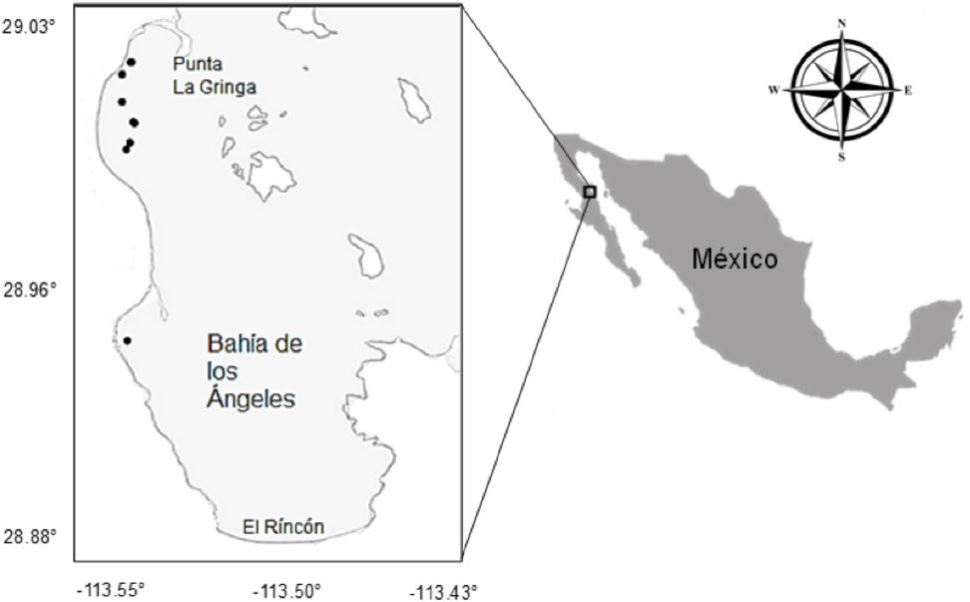
Bahía de Los Ángeles (Gulf of California). The black dots show the sites where the whale shark biopsies (n = 10) were taken (three dots overlap in the sampling area)

Bahía de Los Ángeles (BLA) is located at 28°51′–29°03′ N and 113°26′–113°36′ W (Fig. 1), in Baja California, Mexico. It is a bay with high primary productivity due to an important water exchange with the “Canal de Ballenas” caused by the width of the mouth and the orientation of the bay to the winds, allowing a mixture of warm and cold water. Currents forced by the winds flow through the north channel covering the entire bay almost parallel to the coast (Amador-Buenrostro et al. 1991; Hernández-Nava and Álvarez-Borrego 2013). BLA is a whale shark sighting area where these organisms feed on the surface, mainly due to the presence of copepods (Lavaniegos et al. 2012).

BLA has been declared a priority area for the conservation of biodiversity in the Gulf of California (Coalition for the Sustainability of the Gulf of California 2001) since it is a feeding area for sea turtles, marine mammals, seabirds, and different species of elasmobranchs (Danemann and Ezcurra 2008). The health status of this ecosystem is affected by activities such as tourism and sport, coastal, and industrial fishing (Sáenz-Chávez & Danemann 2008), as well as fuel transportation activities through the Gulf of California to Sonora and Baja California Sur, carried out by a fleet of 12 PEMEX tankers (CONANP-SEMARNAT 2005). The BLA is considered an important area for the development of commercial and sport fishing and water tourism, with two periods of high influx of visitors in April and May; and from June to September (Danemann and Ezcurra 2008).

Skin biopsies from 10 individuals of the species *R. typus* were sampled with collect permit N° SGPA/DGVS/07571/2 at BLA whale shark sighting areas in August 2021, using a Hawaiian-type harpoon with a modified tip disinfected with 70°GL ethanol between samples. Biopsies were taken under the first dorsal fin before the first ridge; on the boat, biopsies were stored in a cooler with ice packs while they were transported to the laboratory. The sex of the sharks was recorded through the presence of claspers in males and their absence in females. Fish size was estimated using an object of known length (in this case, a 25-foot-long boat). Identification photos were taken using a GoPro Black Hero 8 camera, photographing the left side of the organism’s body, specifically enclosing the gills and pectoral fin, to identify the organisms.

To avoid re-sampling the same individual, a PaintSitck All-Weather wax crayon was used to temporarily mark sharks in situ on the first dorsal fin. Whale sharks with lesions were identified by their wounds/scars, so they were not marked. This crayon is not toxic and lasts on the skin of organisms for around 5 days, being deleted after this time.

Biopsies were stored in frozen glass tubes at − 40°C, until their subsequent lyophilization for 24 h. The concentration of 21 individual pesticides (20 OCPs and chlorpyrifos) and 16 individual PAHs (considered as priorities by the US EPA) were determined based on modifications of EPA methods 3550 C, 3535 and 8270D (EPA 2007a, b, c). For each sample, 100–200 mg of lyophilized whale shark skin tissue were extracted twice by ultrasound-assisted extraction (USE) with 12 mL of hexane: dichloromethane (1:1, v/v) and 12 mL of hexane:acetone (1:1, v/v) using an ultrasonic processor (Cole Palmer CPX500) at 50% amplitude over 2 min. After each extraction, the organic phase was separated by centrifugation (5000 rpm for 7 min). Extracts were concentrated using a rotary-evaporator and fractions were separated by solid-phase extraction (SPE) using C-18 500 mg/6mL cartridges (Supelclean ENVI-18, 57,064, Supelco). Cartridges were conditioned with 15 mL of hexane, sample extracts were passed by gravity flow and then eluted with 12 mL of hexane to obtain the aliphatic fraction, and 4 mL of hexane:acetone (9:1, v/v) followed by 4 mL of hexane:dichloromethane (7:3, v/v) and 4 mL of dichloromethane to recover the aromatic fraction. Fractions were evaporated using a gentle nitrogen flow, and individual persistent pollutants were determined by gas chromatography/mass spectrometry (GC-MS) using a gas chromatograph coupled to a mass selective detector operated in electron impact (EI) ionization mode and equipped with an automatic liquid sampler (Agilent Technologies 7890B Series GC; 5977B MSD and 7693 A Autoinjector, respectively). Injections were carried out in split-less mode (1 min) at 280°C. Chromatographic separations were performed using a J&W HP-5MS capillary column (30 m × 0.25 mm and 0.25 μm of film thickness). The carrier gas was He (ultra-pure grade) with a 0.8 mL/min flow rate. For PAHs, the oven temperature was initially set at 60°C, then increased 6°C/min to 290°C (hold time 11.67 min); for OCPs, the initial temperature was 50°C, it was increased 10°C/min to 180°C, then raised 1.5°C/min to 200°C (hold time 2 min), and finally increased 6°C/min to 290°C (hold time 1 min). Mass spectra (m/z 50–550) were recorded at a rate of five scans per second at 70 eV. Mass spectrometric analysis for quantitative determination was performed by selected ion monitoring (SIM Mode) of two characteristic fragment ions for each analyte. Analytical quality control included procedural blanks, calibration curves using analytical standards (Chlorpyrifos PESTANAL® 45395-100MG, Pesticide 8081 Std Mix Supelco CRM46845, QTM PAH Mix Supelco CRM47930), surrogate and internal standards (acenaphthene-d10, phenanthrene-d10 and decachlorobiphenyl). Detection limits for PAHs ranged from 0.1 to 0.8 ng/g, and from 0.6 to 11.1 ng/g for OCPs; recovery percentage of phenanthrene-d10 and decachlorobiphenyl ranged from 65 to 124% (mean ± SD of 98.6% ± 19.9%) and from 68% to 120% (mean ± SD of 97.9% ± 17.7%), respectively; target analytes were not detected in the procedural blanks. In addition to descriptive statistical data of PAH and OCP levels in whale shark skin, a multivariate analysis of the results was performed using the PRIMER 7 (Clarke et al. 2014), including a principal component analysis (PCA), as well as an Analysis of Similarities (ANOSIM) to assess differences between contaminants and the size of whale sharks. The data were analyzed using ordinal nonparametric methods assigning unique ranks to data at and above the detection limit (Helsel 2012). Euclidean distances were used for the resemblance matrix. Only the compounds with more than 30% of samples above the detection limit were used for multivariate analysis.

## Results and Discussion

**Fig. 2:**
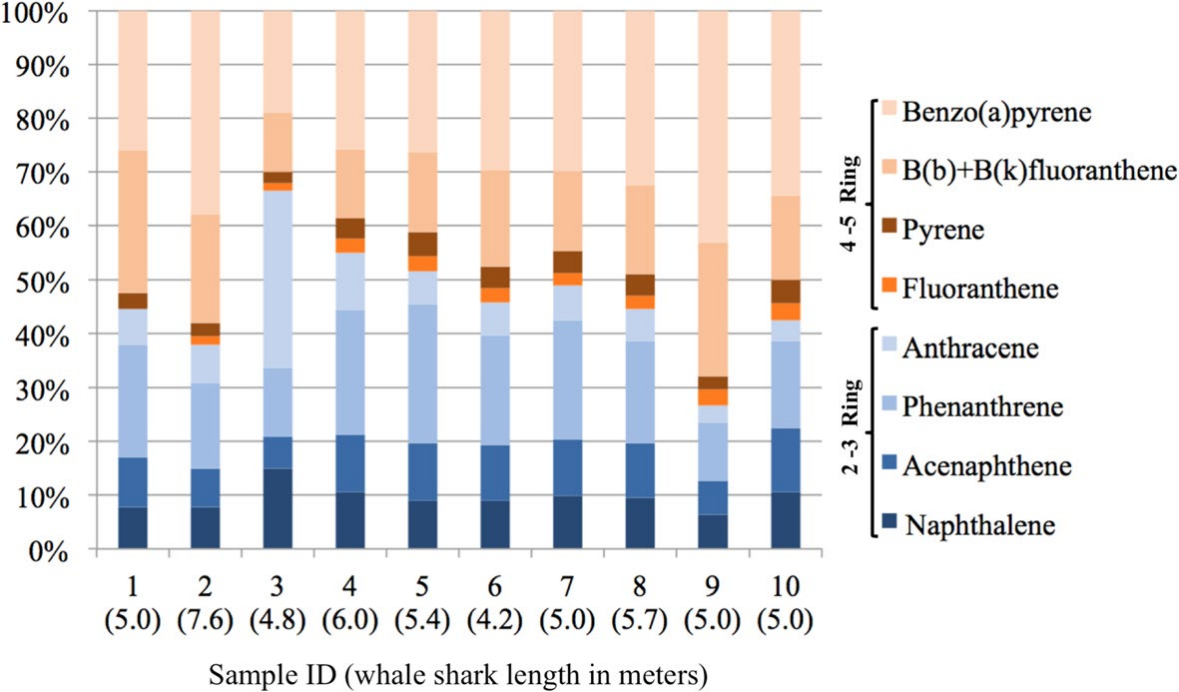
PAH composition in skin biopsies of whale sharks collected in Bahía de los Ángeles

Whale sharks sampled (n = 10) were male and juveniles (4.2 to 7.6 m length); maturity was inferred from length since all individuals were < 8 m (Norman and Stevens 2007), individual lengths are presented in Fig. 2. Descriptive statistics of PAH concentrations in the skin biopsies are presented in Table 1. Total PAHs ranged between of 113.2 and 663.8 ng/g dw, respectively, with 9 of the 16 PAHs analyzed found in the skin of *R. typus*. The average concentration (± one standard deviation) was 279.4 ± 171.4 ng/g dw.

**Table 1:** PAHs (ng/g dw) in whale shark skin from Bahía de Los Ángeles, México (n = 10)

	Mean	Median	Min	Max	IQ Range	SD
Naphthalene (Naph)	26.7	20.0	8.7	71.59	15.9	18.4
Acenaphthene (Ace)	23.8	20.7	10.6	42.19	11.9	9.8
Phenanthrene (Phe)	47.3	38.3	23.6	72.28	36.4	19.0
Anthracene (Ant)	28.5	16.8	7.6	157.34	10.3	45.6
Fluoranthene (Fla)	6.4	5.2	n.d.	19.89	3.3	5.2
Pyrene (Pyr)	9.0	7.8	3.3	16.09	4.9	3.9
Benzo(b) + B(k)fluoranthene (Bb + kFla)	49.4	35.8	21.0	165.02	19.3	42.0
Benzo(a)pyrene (BaP)	88.3	69.8	29.5	286.26	43.4	73.2
Σ PAHs	279.4	221.2	113.2	663.77	161.9	171.4
Σ LMW PAHs	126.3	91.0	50.5	319.74	83.5	79.1
Σ HMW PAHs	153.2	122.6	62.7	487.27	73.0	122.9

Acenaphtylene, fluorene, benzo(a)anthracene, chrysene, indeno(1,2,3-cd)pyrene, dibenzo(a,h)anthracene and benzo(g,h,i)perylene were not detected

*n.d.* not detected, *LMW PAH*s low molecular weight PAHs (Σ 2–3 ring-PAHs), *HMW PAHs* high molecular weight PAHs (Σ 4–5 ring-PAHs), *IQ range* interquartile range

Most of the studies carried out in shark muscle report an average of total PAH levels higher than those detected in this work for *R. typus* in BLA: 5013 ng/g dry weight (dw) in *C. carcharias* (white shark) in South Africa (Marsili et al. 2016); 1330, 1150 and 1080 ng/g wet weight (ww) in *Carcharinus leucas* (bull shark), *C. limbatus* (blacktip shark) and *S. tiburo* (bonnethead shark), respectively, sampled in the northern Gulf of Mexico (Cullen et al. 2019). Those studies were carried out in areas strongly impacted by oil activities (drilling, shipping and/or transportation). Likewise, Al-Hassan et al. (2000) report total PAH concentrations ranging from 150 to 34,840 ng/g ww in the muscle of different shark species from the Arabian Gulf, a coastal ecosystem heavily polluted by petroleum hydrocarbons. Chen et al. (2022) found an average of 234 ng/g ww in silky sharks (*C. falciformis*) collected from the Western Indian Ocean (approximately 836 ng/g dw considering that the authors report an average moisture content in *C. falciformis* muscle of 72%).

The percentage composition of PAHs detected in whale sharks is shown in Fig. 2. Benzo(a)pyrene (BaP) was the most abundant PAH in the skin of whale sharks (19%–43%), except for specimen 3 (4.8 m long), in which anthracene predominated at 33%. The presence of individual PAHs showed the following pattern: BaP > Phe > Bb + kFla > Naph > Ace > Ant > Pyr > Fla. The origin of PAHs in environmental samples (petrogenic vs. pyrogenic) can be assessed by the following ratios (Boehm 1964; Hasanati et al. 2011): LMW/HMW < 1; Phe/Ant < 10; Fla/Pyr > 1 indicate a tendency for PAHs originated by pyrolytic processes. In this study, a slight predominance of HMW PAHs was observed, although higher muscle retention of low molecular weight PAHs has been reported in fish (de Maagd and Vethaak 1998; Marsili et al. 2016; Chen et al. 2022). In addition, the average ratios of Phe/Ant and Fla/Pyr were 3 and 0.6, respectively, that is, a combination of petrogenic and pyrogenic sources. It has been reported that the presence of PAHs in the Gulf of California are associated to local sources such as fishing or tourist boats and fluvial and wind transport of pyrogenic fossil fuels (Páez-Osuna et al. 2017). BaP was the most abundant PAH in the dermal tissue of *R. typus*. This compound is considered carcinogenic, mutagenic, and genotoxic (Gehle 2009). According to the regulations of the European Union (2011), the maximum level allowed in fish muscle is 2.0 ng/g ww. BaP levels found in the whale sharks of BLA exceed this limit (mean = 88.3 ng/g dw); however, it is important to consider that the established values are for muscle, not skin. There are reports of whale shark fishing for human consumption, in the 1990s there was a high demand for whale shark flesh (tofu shark) in Taiwan, India and the Philippines (Rowat and Brooks 2012); likewise, there is evidence that *R. typus* fins and flesh are highly valued in the Chinese market (Li et al. 2012).

Descriptive statistics for pesticide levels in whale shark skin are presented in Table 2. OCPs ranged from 405.6 to 4704.4 ng/g dw, and 14 out of 20 individual OCPs considered for this study were detected. Mean OCP concentration was 1478.2 ± 1247.8 ng/g dw. The pesticides with the highest concentrations (minimum-maximum) were ∑HCHs (204.5–1828.2 ng/g dw), DDT and its metabolites (1.9–1707.1 ng/g dw) and ∑endosulfans (81.4–500.3 ng/g dw).

DDT and its metabolites were detected in the following concentration order: DDT > DDE > DDD, with an average of 303.9 ng/g dw (Table 2); a ratio (p,p′-DDE + p,p′-DDD)/p,p′-DDT lower than 1 suggests that the residues of p,p′-DDT detected in *R. typus* skin biopsies came from “new” sources (Zhang et al. 2018); furthermore, the use of dicofol (a pesticide containing DDT as an impurity) has been reported in Sonora (Rauert et al. 2018), so the proximity of this area to BLA could be a direct source. The presence of heptachlor in the whale shark samples, but the absence of its metabolite (heptachlor epoxide), may also indicate a recent contribution of this pesticide to the area (Granados-Galván et al. 2015); in contrast, the predominance of β-endosulfan over the α-isomer may indicate that there are no recent applications of technical endosulfan in BLA (Jiang et al. 2009). DDT levels were higher in this study than those reported for whale shark skin in Bahía de La Paz (1.3 ng/g ww) (Fossi et al. 2017); the higher levels found in this study can be explained by the difference in units in which the concentrations are expressed (dry weight vs. wet weight), the sampling year (2021 vs. 2014) and the sampling month (August vs. January-February). In specimens of *R. typus* from Djibouti, the presence of DDT and its metabolites (DDE > DDT > DDD) was also reported, but concentration data were not provided (Boldrocchi et al. 2020). In other filter-feeding elasmobranchs, such as the basking shark (*Cethorinus maximus*), a higher mean muscle concentration of ∑DDTs (2001 ng/g lipid weight (lw) has been reported (Fossi et al. 2014); however, it is important to consider the type of tissue (muscle vs. skin) and the units (lipid weight vs. dry weight).

DDT levels found in this study are higher than those reported for hammerhead shark muscle *Sphyrna lewini* in Djibouti (229 ng/g dw), as well as for *S. lewini* and *R. typus* in the Gulf of California with 0.59 and 1.31 ng/g ww, respectively (Fossi et al. 2017; Boldrocchi et al. 2019; Briones et al. 2022). However, DDT concentrations in BLA whale sharks are lower than those found in *C. carcharias* (475.73 ng/g dw), *C. maximus* (2001 ng/g lipid weight (lw) and the Greenland shark (*Somniosus microcephalus*) (1094 ng/g lw) (Fossi et al. 2014; Marsili et al. 2016; Cotronei et al. 2018). This suggests that DDT concentrations vary based on exposure time, geographic distribution, and diet, among other factors (Corsolini et al. 2014; Tiktak et al. 2020). In the case of DDT, a limit of 14.4 ng/g ww has been established for the consumption of this contaminant in fish muscle (EPA 2000). FAO and WHO consider a provisional tolerable daily intake (PTDI) of 10 ng/g of body weight for DDTs (FAO and WHO 2011). In both cases, the concentrations reported in the whale sharks in this study exceed these limits, so consumption of whale shark should be avoided or carried out with caution in areas where people feed on this species. Organochlorine pesticides have already been detected in the Gulf of California, Gutiérrez-Galindo et al. (1992) reported the presence of DDT and its metabolites, heptachlor epoxide, endrin and α-endosulfan. Agrochemicals used in agricultural development areas such as the valley of Mexicali, Sonora and Sinaloa are one of the main sources (Gutiérrez-Galindo et al. 1992; Fossi et al. 2017). In this study, higher concentrations of OCPs than PAHs were found in the skin of whale sharks, contrary to what was reported by Marsili et al. (2016) for great white sharks in South Africa. The great variability of reported concentrations in the different studies suggests a specific accumulation of these contaminants in each species depending on its geographic distribution, trophic level, study tissue, metabolism, and lipid content, as well as the time of exposure to pollutants, growth rate, detoxification capacity, reproductive behavior, opportunistic feeding habits, sex, among other factors (Corsolini et al. 2014; Boldrocchi et al. 2019; Tiktak et al. 2020).

**Table 2:** Pesticides (ng/g dw) in whale shark skin from Bahía de los Ángeles, México (n = 10)

	Mean	Median	Min	Max	IQ Range	SD
α-HCH	112.5	77.4	23.3	448.3	73.1	124.8
β + γ-HCH	576.0	437.6	181.2	1040.3	541.9	309.7
δ-HCH	34.0	n.d.	n.d.	339.6	n.d.	107.4
Aldrin	9.9	n.d.	n.d.	98.8	n.d.	31.2
Heptachlor	174.6	95.3	n.d.	927.9	153.8	276.9
cis-Chlordane	2.2	n.d.	n.d.	8.3	5.0	3.2
p,p-DDE	12.3	13.1	1.9	23.2	9.3	6.2
p,p-DDD	7.5	8.4	n.d.	16.5	13.0	6.3
p,p-DDT	284.1	49.8	n.d.	1667.4	453.9	519.4
Endosulfan I	36.7	25.8	n.d.	96.6	71.0	36.0
Endosulfan II	201.5	208.9	81.4	305.5	171.1	86.0
Endosulfan sulphate	26.5	13.8	n.d.	98.1	38.4	35.4
Methoxychlor	0.3	n.d.	n.d.	3.4	n.d.	1.1
Σ OCPs	1478.2	1102.2	405.7	4704.5	1108.8	1247.8

Heptachlor epoxy, trans-chlordane, dieldrin, endrin, endrin aldehyde, endrin ketone and chlorpyrifos were not detected

*n.d* not detected

**Fig. 3:**
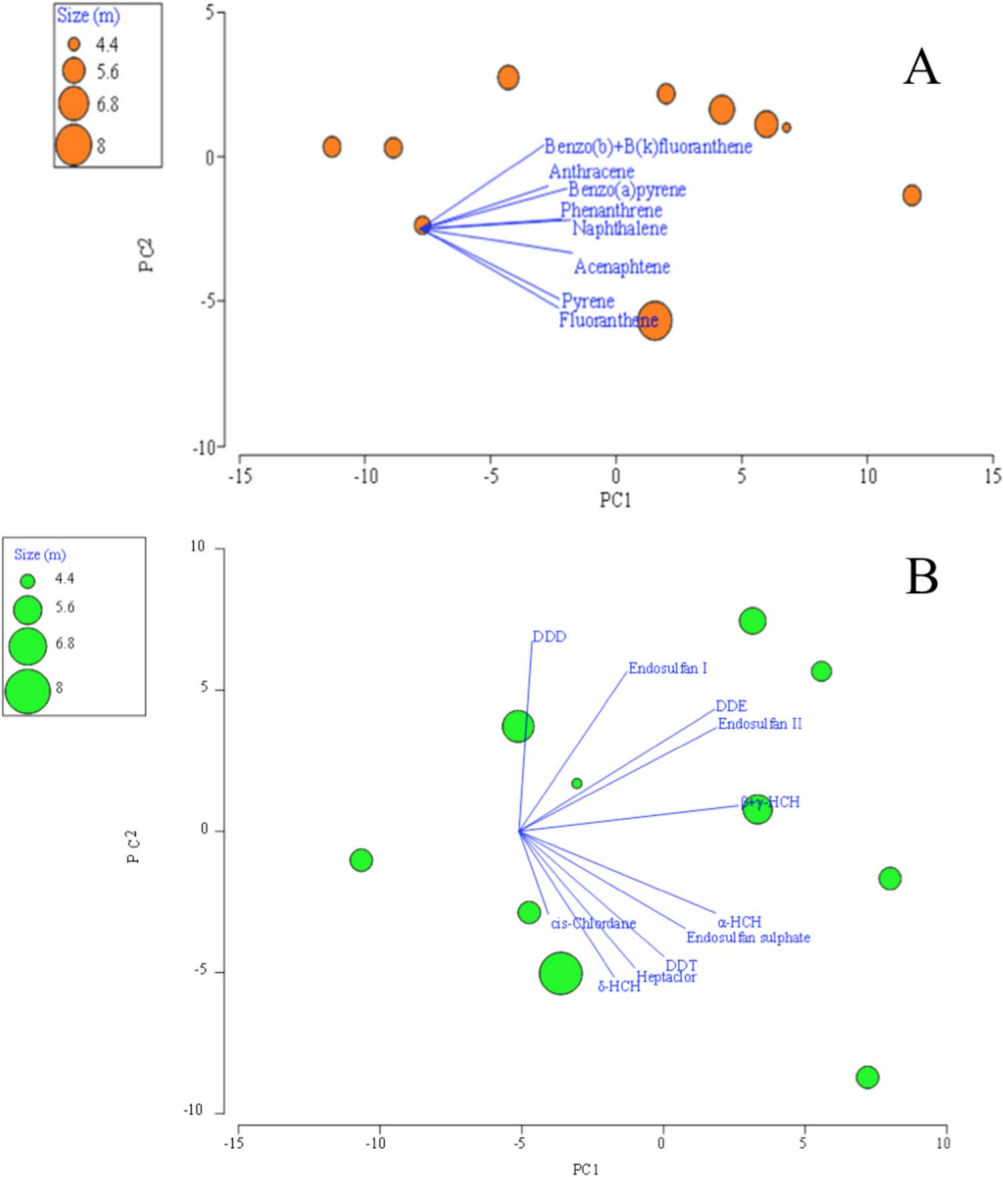
Principal Component Analysis (PCA), representing the size of the whale shark as a variable of interest in relation to the POP concentrations in skin biopsies. **A** PAHs, **B** OCPs

Data obtained were analyzed considering the total length of the whale sharks as a variable. Figure 3 shows the principal component analysis (PCA) related to the size of the organisms and PAH and OCP concentrations (Fig. 3A and B, respectively). Two main components were extracted with an accumulated variance for (A) of 88.8% and 70.2% for (B). When applying the ANOSIM, no significant differences *p* > 5% were found between the size of the whale sharks and the concentrations of PAHs (*p* = 64.8%) and OCPs (*p* = 98.1%). This result may possibly be related to the low variability of sizes, since most of the organisms were between 4.6 and 7.2 m. Similar to this, other reports also showed no significant differences between the concentrations of PAHs with the total length of the organisms (Cullen et al. 2019; Chen et al. 2022). Cagnazzi et al. (2019) also did not found statistically significant relationships between OCP levels and size in species such as the great hammerhead shark (*Sphyrna mokarra*n) and *C. limbatus*. It is important to note that only male and juvenile organisms were sampled, due to the segregation by sex and size of whale sharks in BLA (Eckert and Stewart 2001), so it was not possible to evaluate differences in the content of contaminants with respect to sex; on the other hand, even if *R. typus* is a highly migratory species, they are also considered philopatric organisms, demonstrating fidelity to BLA as a foraging area (Nelson and Eckert 2007; Rowat and Brooks 2012; Ramírez-Macías et al. 2012).

The use of skin to analyze POPs is debatable. The skin is not rich in lipids, unlike the liver that has a high fat content and plays a relevant role in metabolism, detoxification, and accumulation of persistent organic pollutants. However, the skin is a passive sampler for external contamination without the involvement of any metabolic process (Boldrocchi et al. 2022). The dermal denticles are reported to have the ability to easily accumulate particles loaded with contaminants that can be adsorbed on the skin surface, (Corsolini et al. 2014; Boldrocchi et al. 2021). Therefore, the skin could be used as a nondestructive sample that gives information of the concentration of POPs in the whale shark habitat. Also, a positive correlation has been found between POP concentrations in muscle, liver, and subcutaneous tissue in another filter feeding shark, such as the basking shark (Boldrocchi et al. 2022).

In conclusion, data obtained in this study provide relevant information on the presence of persistent organic pollutants in the Gulf of California and, particularly, in a protected and charismatic species, such as the whale shark; information generated using a non-lethal technique based on taking skin biopsies that, even though is not the ideal tissue for the study of POP’s toxicokinetics, it has proven to be a valuable tool for evaluating the exposure to contaminants in the areas where this filter-feeding elasmobranch inhabits.

This work is the first report of the presence of PAHs in whale shark samples from Baja California and Mexico. Benzo(a)pyrene, a hydrocarbon of pyrogenic origin was the PAH found at higher concentrations, above the maximum permitted limit for fish muscle for human consumption. On the other hand, organochlorine pesticides presented higher levels than PAHs in the skin of *R. typus*; ∑HCHs, ∑DDTs, and ∑Endosulfans presented the highest levels. DDT concentrations were higher than those previously reported for whale sharks in the Gulf of California. No significant relationships were found between the levels of PAHs and OCPs and the size of the sharks. The information generated in this work is important for a better management of the species, both for its ecological and economic importance.
